# Use of Early Clinical Trial Data to Support Thorough QT Study Waiver for Upadacitinib and Utility of Food Effect to Demonstrate ECG Assay Sensitivity

**DOI:** 10.1002/cpt.804

**Published:** 2017-09-25

**Authors:** Mohamed‐Eslam F. Mohamed, Jiewei Zeng, Ping Jiang, Balakrishna Hosmane, Ahmed A. Othman

**Affiliations:** ^1^ Clinical Pharmacology and Pharmacometrics, AbbVie Inc North Chicago Illinois USA

## Abstract

Exposure–response analyses of QT data from early‐stage clinical studies represent a valuable tool to assess the QT prolongation potential for drugs in development in lieu of standalone thorough QT (TQT) studies. However, demonstrating adequate electrocardiogram assay sensitivity can be challenging in the absence of a positive pharmacological control. Upadacitinib is a Janus kinase 1 inhibitor currently being evaluated in phase III rheumatoid arthritis trials. Exposure–response analyses to evaluate the QT prolongation potential for upadacitinib from phase I trials and the utility of the effect of food on QTcF to demonstrate ECG assay sensitivity are presented. The analyses demonstrated no effect of upadacitinib on QT interval and confirmed the sensitivity of the ECG assay to detect the small QT shortening effect caused by food. Lack of bias from manual ECG adjudication was also demonstrated. These analyses supported requesting a waiver for the regulatory requirement for a dedicated thorough QT study for upadacitinib.

**Study Highlights**

**WHAT IS THE CURRENT KNOWLEDGE ON THE TOPIC?**

☑ Recent regulatory guidance includes use of exposure–response analysis of early‐stage clinical studies as an approach to evaluate the proarrhythmic potential of investigational compounds as an alternative to conducting a thorough QT (TQT) study.

**WHAT QUESTION DID THIS STUDY ADDRESS?**

☑ The study evaluated the QT prolongation potential for upadacitinib using data from early phase I studies. In the absence of a positive pharmacologic control (e.g., moxifloxacin), the effect of food on the QT interval and bias analysis were used to demonstrate ECG assay sensitivity and to minimize the potential for false‐negative results.

**WHAT DOES THIS STUDY ADD TO OUR KNOWLEDGE?**

☑ Upadacitinib does not prolong the QT interval at the doses being evaluated in patients with rheumatoid arthritis, even under the highest (or worst‐case) potential clinical exposures. Analysis of the food effect on QTcF was successfully used to demonstrate ECG assay sensitivity in early‐stage clinical studies within the context of requesting a waiver for the TQT study requirement in the absence of a positive pharmacologic control.

**HOW THIS MIGHT CHANGE CLINICAL PHARMACOLOGY OR TRANSLATIONAL SCIENCE?**

☑ The proarrhythmic potential of investigational compounds can be evaluated using ECG data collected in early‐phase clinical studies, foregoing the need for a dedicated TQT study. The effect of food on the QT interval can be used as a substitute for a positive pharmacological control to ensure adequate ECG assay sensitivity and can be easily evaluated in early phase I studies. These approaches reduce exposure of healthy volunteers to experimental drugs in dedicated TQT studies and save resources that can be redirected to investigations of other aspects of the efficacy and safety of drug candidates.

Upadacitinib is an oral Janus kinase 1 (JAK1) inhibitor being developed for treatment of several inflammatory diseases including rheumatoid arthritis (RA). Upadacitinib has shown favorable efficacy and acceptable safety profiles^1^, ^2^ and is currently being evaluated in six phase III studies in RA.^3^, ^4^, ^5^, ^6^, ^7^, ^8^ Upadacitinib is also being evaluated in phase II studies in Crohn's disease, ulcerative colitis, and atopic dermatitis and phase III studies in psoriatic arthritis.^9^, ^10^, ^11^, ^12^, ^13^

The 2005 International Conference on Harmonization (ICH) E14 clinical guidance for QT assessment indicates that all new drugs with systemic availability should undergo evaluation of the potential to cause QT prolongation, typically in a thorough QT (TQT) study in healthy subjects.^14^ However, the TQT study is resource‐intensive^15^ and early‐phase clinical studies typically include frequent replicate electrocardiogram (ECG) assessments that can be informative with respect to the potential for a drug in development to cause QT interval prolongation. Recently, the consortium for Innovation and Quality in Pharmaceutical Development and the Cardiac Safety Research Consortium (IQ‐CSRC) reported results from a prospective study supporting the use of exposure–response analysis of QT data collected in early‐phase clinical studies to replace the TQT study.^16^ Consequently, the ICH E14 Guideline Questions and Answers Revision 3 suggested that exposure–response analyses using data acquired from first‐in‐human and/or multiple‐ascending dose (MAD) studies can be used as the primary basis for decisions to classify the risk of QT prolongation of a drug.^17^

One of the requirements for assessment of the QT prolongation potential of drugs, whether through a TQT study or exposure–response analysis of early‐stage clinical studies, is to demonstrate that the ECG assay utilized in the studies of interest had sufficient sensitivity to detect a small change (e.g., mean change of 5 msec) in the QT interval.^14^, ^17^ This is typically demonstrated in TQT studies through administration of moxifloxacin, a drug known to prolong the QT interval by 6–12 msec.^18^ However, administration of moxifloxacin to subjects in early phase I studies can complicate the design and conduct of these studies and may require enrollment of additional subjects. Another approach that may be more feasible to implement in the context of early‐stage clinical studies is to assess the effect of food on the QT interval. Administration of food is known to shorten the Fridericia‐corrected QT interval (QTcF) by 5–10 msec, with the maximum effect occurring ∼2–4 h after a meal.^19^, ^20^, ^21^ Therefore, determination of the effect of food on the QTcF has been proposed as a method to demonstrate ECG assay sensitivity in TQT studies.^19^ However, there are currently no published cases for incorporating the use of food as a positive control within the context of using exposure–response analysis of early‐stage clinical studies to request a waiver of the TQT study regulatory requirement.

One of the other aspects of QT analyses is the use of fully‐automated vs. semiautomated QT measurements. Fully‐automated intervals are measured from the collected ECGs using a computer algorithm with no human intervention. While fully‐automated measurements have the advantage of being consistent and reproducible, they can be subject to errors due to noise or low‐amplitude waves.^17^ To correct such errors, semiautomated measurements are often used where a human reader reviews the fully‐automated measurements and performs adjustments where inaccuracies are observed. Although semiautomated measurement is considered one of the recommended methods by the ICH, it may be subject to bias. Recent analyses by Ferber *et al*. demonstrated that severe bias in the manual correction of QT intervals can result in false‐negative results when evaluating the QT prolongation potential of a drug.^22^ The approach proposed by Ferber *et al*. utilizes the Bland–Altman (BA) slope for the bias in the semiautomated compared to fully‐automated QT measurements. Demonstration of a lack of bias in the semiautomated QT intervals can provide assurance in the results of the exposure–response analyses and protect against false negatives.

In this report, we present exposure–response analyses for the effect of upadacitinib on the QT interval using ECG and plasma concentration data collected from early phase I clinical studies. Additionally, we present an example for demonstrating ECG assay sensitivity and lack of bias within the context of using exposure–response analysis of early‐stage clinical studies in lieu of standalone TQT studies.

## RESULTS

Triplicate 12‐lead ECGs and time‐matched plasma samples were collected from 109 healthy subjects who participated in two phase I clinical studies and received only upadacitinib or placebo (83 received upadacitinib, 26 received placebo).^23^, ^24^ Upadacitinib doses ranged from 1 mg to 48 mg as single doses and from 3 mg to 24 mg twice daily as multiple doses using an immediate‐release formulation. The range of upadacitinib plasma concentrations in the dataset ranged from zero (concentrations below the assay quantitation limit (0.05 ng/mL) were imputed with zero) to 442 ng/mL.

### Model development and evaluation

There was no indication of nonlinearity or hysteresis in the relationship between upadacitinib plasma concentrations and the change from baseline in Fridericia‐corrected QT interval (ΔQTcF) based on exploratory plots (data not shown). Thus, a linear mixed‐effects model was considered appropriate for the analyses (model details are presented in the Methods section).

The model goodness‐of‐fit plots are shown in **Supplemental Figure 1**. No correlation was observed between weighted residuals and the observed ΔQTcF, or between weighted residuals and upadacitinib plasma concentrations. A histogram and a Q‐Q plot of weighted residuals indicated absence of nonnormality in the data (**Supplemental Figure 1**).

### Relationship between upadacitinib plasma concentration and ΔQTcF

**Figure 1:**
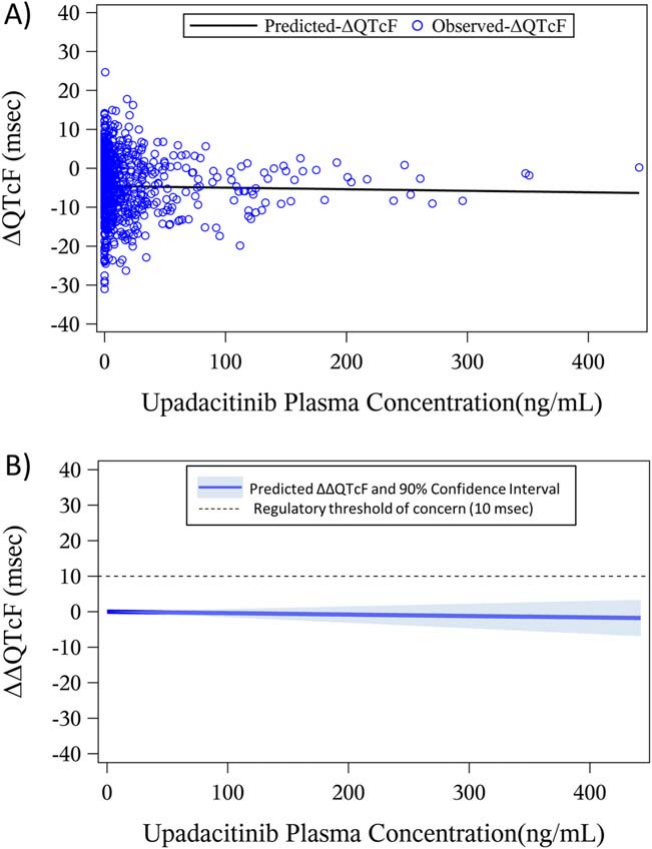
Relationship between (**a**) observed change from baseline in ΔQTcF in the single‐ and multiple‐dose phase I studies vs. upadacitinib plasma concentrations, and (**b**) model‐predicted mean drug effect with two‐sided 90% CI across the range of observed individual upadacitinib plasma concentrations. [Color figure can be viewed at http://wileyonlinelibrary.com]

The relationship between ΔQTcF and upadacitinib plasma concentrations is shown in **Figure** 1 **a** and a summary of the model parameter estimates is presented in **Table** 1. There was no significant relationship between ΔQTcF and upadacitinib plasma concentrations. The estimate for the slope of the relationship was –0.004 msec/ng/mL (*P* = 0.57).

**Table 1:** Definition of parameters and their estimates for the model describing the relationship between ΔQTcF and upadacitinib plasma concentration

Parameter	Estimate (standard error)	*P* value
μ: Intercept (overall mean ΔQTcF; msec)	27.9 (14)	0.04
${\text{β}}_{1}:$ Slope for the effect of baseline QTcF	–0.09 (0.03)	0.01
$\beta_{2}:$ Slope for the effect of upadacitinib concentration (msec/ng/mL)	–0.004 (0.007)	0.57
Random subject effect (ω) variance	22.4	—
Residual error (ɛ) variance	30.6	—

The model‐predicted mean placebo‐corrected ΔQTcF (ΔΔQTcF) and two‐sided 90% confidence intervals (CIs) across the range of observed individual upadacitinib concentrations are presented in **Figure** 1 **b.** Using the nonsignificant slope of an assumed relationship between upadacitinib plasma concentrations and the change from baseline in the QTcF interval, the point estimate of the effect of upadacitinib on ΔΔQTcF was –1.8 msec (upper bound of the 2‐sided 90% confidence bound of 3.3 msec) at the highest individual concentration observed in the analysis dataset (442 ng/mL).

### Demonstration of ECG assay sensitivity through evaluation of the effect of food on QTcF

**Figure 2:**
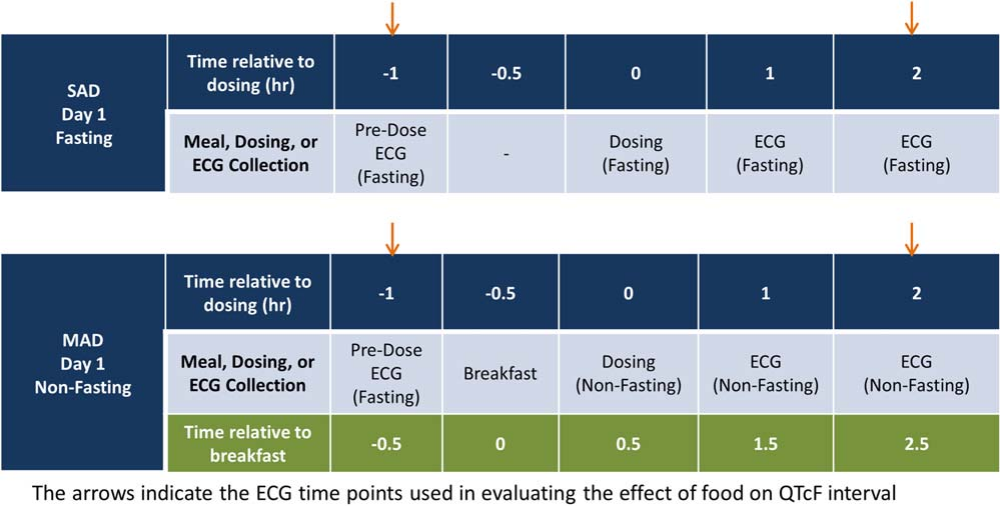
Schematic of the ECG timepoints and meals in the single‐ascending dose (SAD) and multiple‐ascending dose (MAD) studies prior to dosing and in the first 2 h after dosing. [Color figure can be viewed at http://wileyonlinelibrary.com]

The ECG timepoints included in the analysis of the food effect on QTcF are described in the Methods section and are presented in **Figure** 2. At 2 h after placebo dosing (2.5 h after breakfast in the MAD study), the mean (90% CI) ΔQTcF was –3.3 msec (0.1 to –6.7 msec) and –7.7 msec (90% CI: –5.7 to –9.6 msec) in the single‐ascending dose (SAD) (fasting) and MAD (nonfasting) studies, respectively. The time course for the difference in ΔQTcF between subjects who received placebo under nonfasting and fasting conditions is provided in **Supplemental Figure 2**. Analysis of covariance (ANCOVA) of QTcF for the baseline and 2‐h postdose (2.5 h after breakfast) timepoints, including fasting status as the main factor, demonstrated that food shortened the QTcF by a mean of 5.9 msec (2‐sided 90% CI: –9.6 to –2.1 msec), which is within the range of the expected effect of food on QTcF previously reported.^19^, ^20^, ^21^, ^25^ These results demonstrate that the ECG assay had adequate sensitivity to detect the small change in QTcF caused by food.

### Assessment of bias in semiautomated QTcF measurements

**Figure 3:**
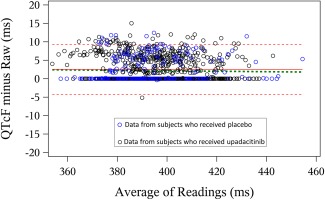
Assessment of bias in QTcF measurements. Bland–Altman plot for the full analysis set comparing the semiautomated and fully‐automated QTcF measurements. Footnote: Red solid and dotted lines represent mean ± 2 standard deviations of the difference between the two measurement methods; green dashed line represents regression through the data using an M estimator as described by Ferber *et al*.^22^ [Color figure can be viewed at http://wileyonlinelibrary.com]

Semiautomated QTcF measurements were compared to fully‐automated QTcF measurements using BA plots and the BA slope was estimated using robust regression as previously described.^22^ The BA plot for the assessment of bias is shown in **Figure** 3. Additionally, the analysis was performed separately on two subsets: on subjects who received upadacitinib and on subjects who received placebo (**Table** 2).

**Table 2:** Summary of BA plot parameters comparing the fully‐automated and semiautomated QTcF measurements

Analysis set	Difference, semiautomated minus fully automated, mean (95% CI), msec	BA slope, mean (95% CI), msec^a^
Full analysis set (*n* = 109)	2.52 (2.31 to 2.73)	–0.6 (–2.0 to 0.8)
Upadacitinib analysis set (*n* = 83)	2.57 (2.33 to 2.81)	–1.8 (–3.4 to –0.2)
Placebo analysis set (*n* = 26)	2.35 (1.91 to 2.79)	2.2 (–0.4 to 4.7)

a Presented as milliseconds over a QTcF range of 100 milliseconds.

For the full analysis set and each of the analysis subsets, the BA slopes were below the prespecified cutoff of –10 msec/100 msec, indicating lack of measurement bias. The mean difference in QTcF intervals between the semiautomated and the fully‐automated ECG measurements was less than 3 msec. It is worth noting that the mean difference in all datasets was positive, indicating that manual adjudication of the QT measurements by the over‐reader tended to correct in the direction of slightly prolonging QT intervals compared to the fully‐automated measurements. There was no statistically significant difference in the degree of positive correction for the upadacitinib and placebo Analysis Sets (2.6 msec vs. 2.4 msec).

## DISCUSSION

The presented exposure–response analyses indicate a lack of effect of upadacitinib on the QT interval at plasma concentrations exceeding the predicted supratherapeutic concentrations of upadacitinib in patients with RA. These analyses, supported by adequate ECG assay sensitivity and a lack of bias in QTcF measurements, were accepted by the US Food and Drug Administration, the European Medicines Agency, and the Japan Pharmaceuticals and Medical Devices Agency in lieu of a thorough QT study to support the development of upadacitinib in RA, assuming no future safety concerns are raised based on new safety information.

The studies included in the exposure–response analyses encompassed a wide range of single (1–48 mg) and multiple (3–24 mg twice daily) doses of upadacitinib immediate‐release capsule formulation. The highest upadacitinib plasma concentration observed in these studies was 442 ng/mL (**Figure** 1). At this high concentration, the predicted effect of upadacitinib on QTcF was well below the regulatory threshold of concern of 10 msec, indicating a lack of QT prolongation potential. Upadacitinib is being administered in the ongoing phase III studies in RA as an extended‐release formulation at doses of 15 mg and 30 mg once daily^3^, ^4^, ^5^, ^6^, ^7^, ^8^ The 30 mg once daily dose of upadacitinib using the extended‐release formulation is predicted to have a mean C_max_ of ∼100 ng/mL. Upadacitinib is a nonsensitive substrate for metabolism through CYP3A enzymes and ∼20% of upadacitinib dose is eliminated unchanged in urine. Strong CYP3A inhibition by ketoconazole increased upadacitinib exposures by ∼70%.^24^ Clinical studies are currently ongoing to characterize the effect of renal and hepatic impairment on upadacitinib pharmacokinetics. However, given that there are multiple routes of elimination for upadacitinib, exposures in subjects with renal and hepatic impairment are expected to be within 2‐fold of the exposures in subjects with normal renal and hepatic function. Therefore, the range of concentrations evaluated in the exposure–response analysis encompasses the supratherapeutic exposures under strong CYP3A inhibition and the exposures that may be observed in subjects with hepatic or renal impairment (mean C_max_ of ∼200 ng/mL under a worst‐case scenario). In the absence of QT evaluations at concentrations that are at least 3‐fold the predicted supratherapeutic concentrations in patients, it was necessary to demonstrate adequate ECG assay sensitivity to assure lack of potential false‐negative results.^26^

To demonstrate assay sensitivity in a TQT study, moxifloxacin is typically used as a positive control because it prolongs QTcF by ∼10 msec.^18^ However, inclusion of moxifloxacin in early‐stage clinical studies can complicate the study design and may require additional subjects or treatment periods. In this study, we utilized the QTcF shortening effect of food to successfully demonstrate ECG assay sensitivity.^18^ Evaluating the effect of food on QTcF to demonstrate assay sensitivity in early‐stage clinical studies is an attractive option, particularly when sufficiently high multiples (e.g., 3‐fold) of the highest clinically relevant concentrations (e.g., concentrations under the effect of enzyme inhibitors) have not been achieved in those early studies.^27^ Many of the early‐stage clinical studies include administration of the drug of interest under fasting conditions and under well‐controlled fed conditions that ensure consistency of the caloric intake and timing of meals relative to study activities. Inclusion of a fasting cohort, as in the presented analysis, enables a more robust assessment of the food effect than having only a fed cohort, as it accounts for differences in QTcF due to the time of day. In the presented analyses, estimating the effect of food in the nonfasting cohort as a change from baseline (with no fasting control) slightly overestimated the QTcF shortening by food compared to inclusion of the fasting cohort (–7.7 vs. –5.9 msec).

Another approach to increase confidence in the results of exposure–response analyses of early‐stage clinical studies is to evaluate the bias in semiautomated compared to fully‐automated QTcF measurements through BA plots, which display the QTcF differences between the two methods vs. the QTcF means of the two methods.^22^ Through this approach, the BA slope is estimated and can be used to evaluate bias in the manual correction of QTcF measurements. Simulations of different magnitudes of bias conducted by Ferber *et al*.^22^ demonstrated that a BA slope of less than 10 msec/100 msec is unlikely to result in a false‐negative classification of the QT prolongation risk. In the current study, the BA slope was estimated to be well below that limit, indicating lack of bias and providing further assurance that upadacitinib has no potential to prolong QTcF at therapeutic or supratherapeutic plasma concentrations.

In summary, the presented analyses demonstrated a lack of QT prolongation potential for upadacitinib at the doses being used in RA phase III studies. Additionally, the effect of food on QTcF was used to successfully demonstrate ECG assay sensitivity. Analysis of bias was conducted to provide further confidence in the results of the exposure–response analyses. Use of food to demonstrate ECG assay sensitivity and analysis of bias can both be easily implemented within early‐stage phase I clinical trials to minimize the potential for false‐negative QT prolongation conclusions. These analyses described herein supported a request for a regulatory waiver of the TQT study requirement for development of upadacitinib in RA.

## METHODS

### Participants and study designs

The analysis dataset included triplicate 12‐lead ECGs and time‐matched upadacitinib plasma concentrations from healthy adult subjects who participated in two phase I clinical studies. A summary of the number of subjects included in the analysis by study and dosing regimen is provided in the **Supplemental Table.** The details of the studies have been previously reported.^23^, ^24^ Study 1 included two substudies. Substudy 1 was a SAD substudy, which was designed as a randomized, double‐blind, placebo‐controlled study in which healthy adult participants received a single oral dose of upadacitinib immediate‐release capsules (1, 3, 6, 12, 24, 36, or 48 mg) or placebo in the morning after a 10‐h fast.^23^ Substudy 2 was an evaluation of the effect of food and ketoconazole on upadacitinib pharmacokinetics. Substudy 2 was an open‐label, randomized, two‐sequence, crossover evaluation in which a single oral dose of 3 mg upadacitinib was administered on three different occasions: in the morning after a 10‐h overnight fast; 30 min after starting a high‐fat breakfast; and in the morning on Day 4 of a 6‐day regimen of once‐daily ketoconazole.^24^ For all fasting regimens in Study 1, subjects fasted for an additional 4 h after the dose was administered. Data from Substudy 2 from the period in which subjects received ketoconazole were not included in the analysis to avoid any potential confounding effect on QTcF by ketoconazole.

Study 2 was designed as a randomized, double‐blind, placebo‐controlled study in which healthy adult participants received twice‐daily doses of upadacitinib immediate‐release capsules (3, 6, 12, and 24 mg) or placebo for 13 days and once in the morning of Day 14.^23^ Upadacitinib was administered ∼30 min after a standard breakfast or an evening snack. Study 2 also included a substudy in subjects with RA. This substudy was not included in the analysis to ensure homogeneity of the subjects being evaluated (all healthy subjects) and to avoid potential interference from concomitant medications. Both studies were conducted according to good clinical practice guidelines and the ethical principles that have their origin in the Declaration of Helsinki. The study protocols were approved by the Institutional Review Boards and all subjects provided written informed consent.

### ECG assessments

The analysis dataset included triplicate 12‐lead ECGs collected at 12 h before dosing on Day 1 in addition to predose and 1, 2, 6, and 12 h postdose on Day 1 of Study 1 and on Days 1 and 14 of Study 2. The electronic tracings of all ECGs performed in triplicate were transferred to and evaluated by eECG/ABBIOS (electronic ECG/ABbvieBIOsignal System), which used a validated automated signal analysis algorithm to measure predefined ECG intervals (RR, PR, QT, and QRS duration). A qualified over‐reader reviewed the electronic ECG data using standardized quality‐review criteria.

The semiautomated QT interval measurements were used in the analyses. The corrected QT interval (QTc) was calculated using Fridericia's correction method, which was applied to the raw QT interval data prior to any transformation. QTcF is the value of QTc obtained using the Fridericia correction formula.^14^, ^28^

The change in QTcF from baseline (ΔQTcF) was calculated as:

$$\Delta QTcF_{i,t}=QTcF_{i,t}-QTcF_{i,Baseline}$$

where ΔQTcF for individual i at time t was calculated as the difference between the QTcF values for the individual at time t and at baseline. The triplicate QTcF values at each postdose timepoint were averaged, and the resulting single mean value was used to obtain ΔQTcF at each timepoint for each individual for subsequent analyses. Individual placebo‐adjusted ΔQTcF (ΔΔQTcF) values were computed as ΔQTcF minus the time‐matched mean ΔQTcF of the placebo group.

### Pharmacokinetic sampling

Serial blood samples were collected over 72 h after single dosing (Study 1) or over 12 h after the first dose and over 72 h after the last dose of multiple dosing (Study 2).^23^, ^24^ Plasma concentrations of upadacitinib were included in the analysis dataset if the timing of the blood sample coincided with an ECG measurement (predose and 1, 2, 6, and 12 h postdose).

### Exposure–response analysis

To evaluate the risk of upadacitinib QT interval prolongation, a linear mixed‐effects exposure–response analysis was conducted using SAS software (v. 9.3; SAS Institute, Cary, NC). For the analysis of ΔQTcF, the baseline value was defined as the average of the predose values obtained ∼12 h prior to dosing on Day –1 and immediately prior to dosing on Day 1.

All available data were included in the analysis and all below‐the‐limit of quantitation concentrations and the concentration values for placebo subjects were set to zero. The presence of hysteresis was evaluated by investigating plots of upadacitinib concentration and ΔΔQTcF vs. time and hysteresis loop plots, stratified by upadacitinib dose.

The linear mixed effects model was given by:

$$\Delta QTcF=\mu +\omega +\beta_{1}*baseline+\beta_{2}*concentration+TimeDayStudyFactor+\varepsilon$$

where μ is the overall mean of the response ΔQTcF; β_1_ is the slope for the effect of QTcF baseline value on ΔQTcF; β_2_ is the slope for the effect of upadacitinib concentration on ΔQTcF; and the TimeDayStudyFactor represents combined effects of time, day, study, and their interactions; the term ε is the residual and is assumed to be independently and identically normally distributed with mean 0 and variance $\sigma_{\omega }^{2}$. The random subject effect ω, which induces compound symmetry structure for all the data within a subject, is assumed to be independent of ε and also assumed to be independently and identically normally distributed with mean 0 and variance $\sigma_{\omega }^{2}$. The covariates of age, sex, race, and body mass index (BMI) were included initially in the model and were excluded using a backward elimination method. The covariates that were not significant at a level of 0.1 were excluded from the model.

### ECG assay sensitivity analysis using the effect of food

In the SAD and MAD studies, a triplicate ECG was collected under fasting conditions at ∼1 h before dosing placebo or upadacitinib, which corresponded to 30 min before breakfast in the MAD study, and at 2 h after dosing, which corresponded to 2.5 h after breakfast in the MAD study (**Figure** 2). Subjects in the SAD study continued to fast after dosing for an additional 4 h. The ECG collected at 2.5 h after breakfast was within the time window for the maximum effect of a meal on QTcF (2 to 4 h after a meal). Subjects from the SAD study who had the 2‐h postdose ECG collected under fasting conditions were included in the analysis to account for the effect of time of day on QTcF.

The analysis dataset included triplicate 12‐lead ECGs collected from all subjects who received placebo and had QT measurements at predose baseline and 2 h after dosing (23 subjects). To estimate the effect of food on QTcF, the change in QTcF from baseline to 2 h postdose was compared between the fasting and nonfasting groups using ANCOVA, with fasting condition as a main factor and baseline QTcF and sex as covariates. Baseline QTcF was defined as the predose measurement on Day 1. The estimate of food effect on QTcF (nonfasting minus fasting) and the 2‐sided 90% CI were calculated.

### Analysis of bias evaluation in QTcF measurements

Bias analysis was performed on the Full Analysis Set (all subjects with measurements included in the exposure–response analysis) in addition to separate analyses on the subset of subjects who received upadacitinib and those who received placebo. The mean QTcF value of replicates at each timepoint was analyzed according to the methodology specified in Ferber *et al*.^22^ For each subject, the QTcF values measured at each timepoint with the fully‐automated and semiautomated methods were compared using a BA plot, which displays the QTcF differences vs. the QTcF means of the two methods. The relationship between the means and differences of the two methods was explored by robust regression analysis using an M estimation. From the model, the fitted slope and associated 2‐sided 95% CIs were provided.

## CONFLICT OF INTEREST/DISCLOSURE

All authors are employees or contractors of AbbVie and hold AbbVie stock or stock options.

## AUTHOR CONTRIBUTIONS

M.F.M., J.Z., and A.A.O. wrote the article; M.F.M. and A.A.O. designed the research; M.F.M., J.Z., P.J., B.H., and A.A.O. performed the research; J.Z., P.J., and B.H. analyzed the data.

## Supporting information


**SUPPLEMENTARY MATERIAL** is linked to the online version of the article at http://www.cpt-journal.com

Supporting Information

Supporting Information

Supporting Information
