# Supplementary material for: Use of Early Clinical Trial Data to Support Thorough QT Study Waiver for Upadacitinib and Utility of Food Effect to Demonstrate ECG Assay Sensitivity
Source: Clin Pharmacol Ther. 2017 Sep 25;103(5):836–42. doi: 10.1002/cpt.804 (PMC5946993; doi:10.1002/cpt.804)

Supplemental Figure 1. Diagnostic Plots for the Goodness of Fit of the Exposure-Response Model Characterizing the Effect of Upadacitinib on ΔQTcF

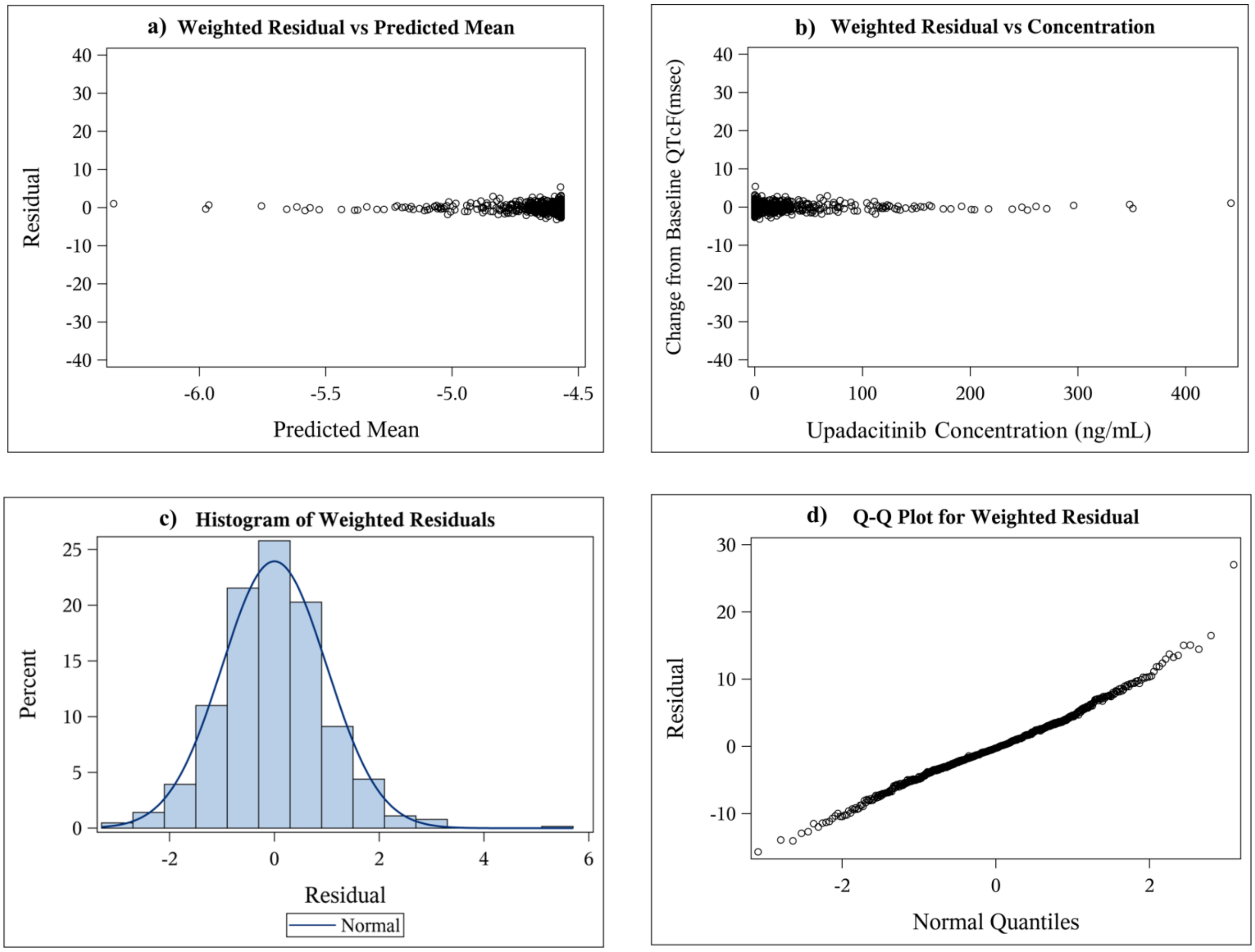

Supplement: Supplementary file 1 — Supporting Information [file CPT-103-836-s001.pdf]
