# Supplementary material for: Use of Early Clinical Trial Data to Support Thorough QT Study Waiver for Upadacitinib and Utility of Food Effect to Demonstrate ECG Assay Sensitivity
Source: Clin Pharmacol Ther. 2017 Sep 25;103(5):836–42. doi: 10.1002/cpt.804 (PMC5946993; doi:10.1002/cpt.804)

Supplemental Figure 2. Effect of Food on QTc Interval

Data points represent the ANCOVA-based difference (and 90% confidence interval) in ΔQTcF between subjects who received placebo under non-fasting (MAD study) and fasting (SAD study) conditions. Subjects received placebo 30 minutes after breakfast in the MAD study and under fasting conditions in the SAD study and continued to fast for 4 hours after dosing. Subjects received lunch 4 hours after dosing in both of the studies, which explains the lack of difference between the two cohorts after 2 hours (grey symbols and dashed line).

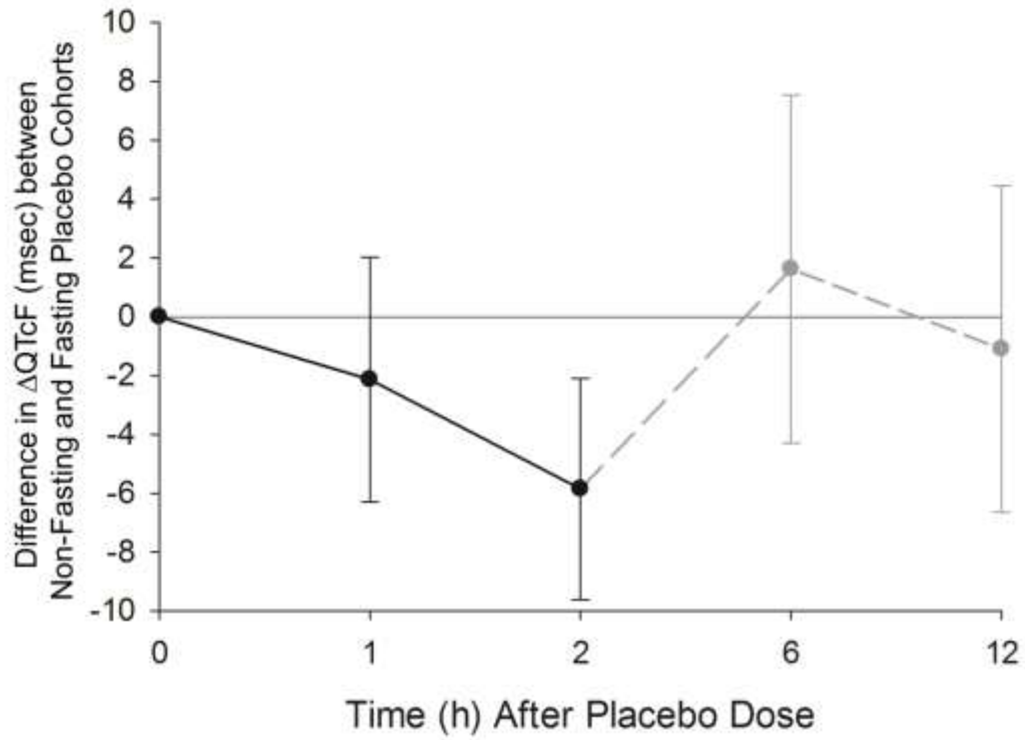

Supplement: Supplementary file 2 — Supporting Information [file CPT-103-836-s002.pdf]
