# Supplementary material for: Use of Early Clinical Trial Data to Support Thorough QT Study Waiver for Upadacitinib and Utility of Food Effect to Demonstrate ECG Assay Sensitivity
Source: Clin Pharmacol Ther. 2017 Sep 25;103(5):836–42. doi: 10.1002/cpt.804 (PMC5946993; doi:10.1002/cpt.804)
Supplement: Supplementary file 3 — Supporting Information [file CPT-103-836-s003.pdf]

Supplemental Table. Number of Subjects Included in the Exposure-Response Analysis by Study and Regimen

Study	Regimen	Number of subjects
Study 1 (Single ascending dose substudy)	1 mg upadacitinib (fasting)	6
	3 mg upadacitinib (fasting)	6
	6 mg upadacitinib (fasting)	5[b]
	12 mg upadacitinib (fasting)	6
	24 mg upadacitinib (fasting)	5[b]
	36 mg upadacitinib (fasting)	5[b]
	48 mg upadacitinib (fasting)	6
	Placebo (fasting)	14
Study 1 (Single-dose food effect and ketoconazole substudy)[a]	3 mg upadacitinib	12
Study 2 (Part 1 multiple-dose study)	3 mg upadacitinib BID (nonfasting)	8
	6 mg upadacitinib BID (nonfasting)	8
	12 mg upadacitinib BID (nonfasting)	8
	24 mg upadacitinib BID (nonfasting)	8
	Placebo (nonfasting)	12
		Total = 109

a. Conducted in 12 subjects using a 3-period cross-over design. Data from the period in which subjects received ketoconazole were not included in the analyses.

b. Total number enrolled in each of the noted dose groups was 6. One subject enrolled in each of these dose groups had a missing baseline ECG and was not included in the analyses.
